# Dichlorido[(*S*)-(1-phenyl­ethyl)(2-pyridyl­meth­yl)amine-κ^2^ *N*,*N*′]zinc(II)

**DOI:** 10.1107/S1600536808003541

**Published:** 2008-02-06

**Authors:** Quang Trung Nguyen, Jong Hwa Jeong

**Affiliations:** a Department of Chemistry, Kyungpook National University, Taegu 702-701, Republic of Korea

## Abstract

In the title compound, [ZnCl_2_(C_14_H_16_N_2_)], the Zn^II^ atom is coordinated by two N atoms and two Cl atoms in an approximately tetra­hedral arrangement. The dihedral angle between the N—Zn—N and Cl—Zn—Cl planes is 88.06 (8)°. The H atoms on the chiral C atom and the adjacent N atom have an *anti* conformation.

## Related literature

For the synthesis of (*S*)-2-pyridinal-1-phenyl­ethyl­imine, see: Kang *et al.* (2006 ▶). For related structures, see: Moreau *et al.* (1999 ▶); Mizushima *et al.* (1999 ▶); Himeda *et al.* (2003 ▶).
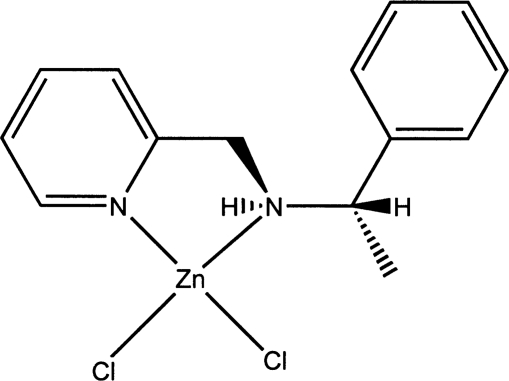


## Experimental

### 

#### Crystal data

[ZnCl_2_(C_14_H_16_N_2_)]
*M* *_r_* = 348.56
Orthorhombic, 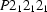

*a* = 9.2342 (6) Å
*b* = 12.5782 (10) Å
*c* = 13.4032 (8) Å
*V* = 1556.78 (18) Å^3^
*Z* = 4
Mo *K*α radiation
μ = 1.91 mm^−1^
*T* = 293 (2) K
0.40 × 0.40 × 0.30 mm

#### Data collection

Enraf–Nonius CAD-4 diffractometer
Absorption correction: ψ scan (*ABSCALC*; McArdle & Daly, 1999 ▶) *T*_min_ = 0.485, *T*_max_ = 0.564
1705 measured reflections
1659 independent reflections
1530 reflections with *I* > 2σ(*I*)
*R*_int_ = 0.009
3 standard reflections frequency: 60 min intensity decay: 0.2%

#### Refinement

*R*[*F* ^2^ > 2σ(*F* ^2^)] = 0.030
*wR*(*F* ^2^) = 0.080
*S* = 1.07
1659 reflections
173 parameters
H-atom parameters constrained
Δρ_max_ = 0.56 e Å^−3^
Δρ_min_ = −0.57 e Å^−3^
Absolute structure: Flack (1983 ▶), 2 Friedel pairs
Flack parameter: 0.018 (19)

### 

Data collection: *CAD-4 Software* (Enraf–Nonius, 1989 ▶); cell refinement: *CAD-4 Software*; data reduction: *XCAD* (McArdle, 1999 ▶); program(s) used to solve structure: *SHELXS97* (Sheldrick, 2008 ▶); program(s) used to refine structure: *SHELXL97* (Sheldrick, 2008 ▶); molecular graphics: *ORTEXIII* (McArdle, 1995 ▶); software used to prepare material for publication: *SHELXL97*.

## Supplementary Material

Crystal structure: contains datablocks global, I. DOI: 10.1107/S1600536808003541/cf2180sup1.cif

Structure factors: contains datablocks I. DOI: 10.1107/S1600536808003541/cf2180Isup2.hkl

Additional supplementary materials:  crystallographic information; 3D view; checkCIF report

## Figures and Tables

**Fig. 1.**
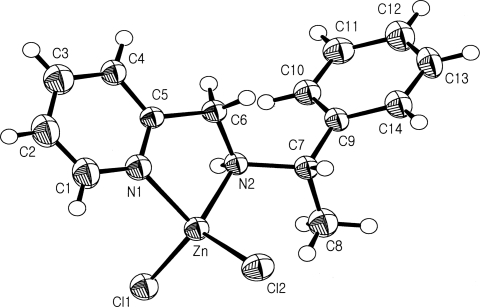
The molecular structure. Displacement ellipsoids are drawn at the 40% probability level.

## supplementary crystallographic information

### Comment

The ligand, (*S*)-(1-Phenylethyl)(2-pyridylmethyl)amine, was obtained from reduction of (*S*)-2-pyridinal-1-phenylethylimine (Kang *et al.*, 2006) with NaBH_4_ in methanol solution. The ligand was used as co-ligand with another chiral ligand in Ru or Rh complexes as the catalyst for hydrogenation of ketones (Moreau *et al.*, 1999; Mizushima *et al.*, 1999; Himeda *et al.*, 2003). In the crystal structure, the geometry around the Zn^II^ ion is approximately tetrahedral with bonds being formed by two chloride ions and the pyridyl and amine nitrogen atoms of the ligand (Fig. 1). The dihedral angle between the N—Zn—N and Cl—Zn—Cl planes is 88.06 (8)°. The H atoms on the chiral carbon atom and the adjacent nitrogen atom have an anti conformation.

### Experimental

NaBH_4_ (0.33 g, 8.8 mmol) was added slowly to a solution of (*S*)-2- pyridinal-1-phenylethylimine (1.79 g, 8.5 mmol) in methanol (15 ml). The mixture was stirred overnight, and the solvent was removed by evaporation. The residue obtained was dissolved in 20 ml distilled water and the organic product was extracted with CH_2_Cl_2_ (3 *x* 20 ml) and dried over anhydrous MgSO_4_. The solvent was evaporated to give a pale yellow oil; 1.41 g (78% yield). ^1^H-NMR (400 MHz, CDCl_3_) δ 7.39 (t, 1H, ArH), 7.26 (m, 4H, ArH), 7.17 (m,1*H*, ArH), 6.90 (t, 2H, ArH), 3.74 (q, J=6.56 Hz, 1H, CH), 3.59 (s, 2H, CH_2_), 2.45 (s, 3H, PyCH_3_), 2.19 (br, s, 1H, NH), 1.30 (d, J=6.56 Hz, 3H, CH_3_). A solution of the ligand (0.96 g, 4.5 mmol) in ethanol (5 ml) was added dropwise to a solution of ZnCl_2_ (0.61 g, 4.5 mmol) in ethanol (10 ml). The mixture was stirred overnight at room temperature. The solvent was removed to yield a white solid product. Colorless crystals were obtained by slowly diffusing diethyl ether into a saturated solution in acetonitrile (1.36 g, 87%). Anal. Calcd. for C_14_H_16_Cl_2_N_2_Zn: C, 48.23; H, 4.63; N, 8.04. Found: C, 48.19; H, 4.70, N, 8.01%. ^1^H-NMR (400 MHz, CD_3_CN) δ 7.89 (m, 1H, ArH), 7.44 (m, 6H, ArH), 7,16 (d, J=7.79 Hz, 1H, ArH), 4.15 (m, 2H, NH & CH), 3.77 (m, 2H, CH_2_), 2.78 (s, 3H, PyCH_3_), 1.70 (d, J=3.24 Hz, CH_3_).

### Refinement

All H atoms were positioned geometrically and refined using a riding model, with C—H = 0.93 Å, *U*_iso_(H) = 1.2*U*_eq_(C) for C(*sp*^2^)H, C—H = 0.97 Å, *U*_iso_(H) = 1.2*U*_eq_(C) for CH_2_, C—H = 0.96 Å, *U*_iso_(H) = 1.5*U*_eq_(C) for CH_3_, and N—H = 0.91 Å, *U*_iso_(H) = 1.2*U*_eq_(N) for NH atoms.

### Crystal data

**Table:**

[ZnCl_2_(C_14_H_16_N_2_)]	*F*_000_ = 712
*M**_r_* = 348.56	*D*_x_ = 1.487 Mg m^−^^3^
Orthorhombic, *P*2_1_2_1_2_1_	Mo *K*α radiation λ = 0.71073 Å
Hall symbol: P 2ac 2ab	Cell parameters from 25 reflections
*a* = 9.2342 (6) Å	θ = 9.9–13.0º
*b* = 12.5782 (10) Å	µ = 1.91 mm^−^^1^
*c* = 13.4032 (8) Å	*T* = 293 (2) K
*V* = 1556.78 (18) Å^3^	Block, colorless
*Z* = 4	0.40 × 0.40 × 0.30 mm

### Data collection

**Table:**

Enraf–Nonius CAD-4 diffractometer	*R*_int_ = 0.009
Radiation source: fine-focus sealed tube	θ_max_ = 25.5º
Monochromator: graphite	θ_min_ = 2.2º
*T* = 293(2) K	*h* = 0→11
ω/2θ scans	*k* = −14→0
Absorption correction: ψ scan(ABSCALC; McArdle & Daly, 1999)	*l* = 0→15
*T*_min_ = 0.485, *T*_max_ = 0.564	3 standard reflections
1705 measured reflections	every 60 min
1659 independent reflections	intensity decay: 0.2%
1530 reflections with *I* > 2σ(*I*)	

### Refinement

**Table:**

Refinement on *F*^2^	Hydrogen site location: inferred from neighbouring sites
Least-squares matrix: full	H-atom parameters constrained
*R*[*F*^2^ > 2σ(*F*^2^)] = 0.030	*w* = 1/[σ^2^(*F*_o_^2^) + (0.0598*P*)^2^ + 0.2097*P*] where *P* = (*F*_o_^2^ + 2*F*_c_^2^)/3
*wR*(*F*^2^) = 0.080	(Δ/σ)_max_ < 0.001
*S* = 1.07	Δρ_max_ = 0.56 e Å^−^^3^
1659 reflections	Δρ_min_ = −0.57 e Å^−^^3^
173 parameters	Extinction correction: none
Primary atom site location: structure-invariant direct methods	Absolute structure: Flack (1983), 2 Friedel pairs
Secondary atom site location: difference Fourier map	Flack parameter: 0.018 (19)

### Special details

**Table:**

Geometry. All e.s.d.'s (except the e.s.d. in the dihedral angle between two l.s. planes) are estimated using the full covariance matrix. The cell e.s.d.'s are taken into account individually in the estimation of e.s.d.'s in distances, angles and torsion angles; correlations between e.s.d.'s in cell parameters are only used when they are defined by crystal symmetry. An approximate (isotropic) treatment of cell e.s.d.'s is used for estimating e.s.d.'s involving l.s. planes.

### Fractional atomic coordinates and isotropic or equivalent isotropic displacement parameters (Å^2^)

**Table:**

	*x*	*y*	*z*	*U*_iso_*/*U*_eq_	
Zn	0.04578 (5)	0.93541 (3)	0.73653 (3)	0.04068 (15)	
Cl1	−0.15959 (12)	0.94695 (8)	0.65366 (7)	0.0527 (3)	
Cl2	0.14716 (13)	1.08625 (8)	0.78507 (9)	0.0584 (3)	
N1	0.1861 (4)	0.8349 (3)	0.6668 (2)	0.0464 (8)	
N2	0.0324 (3)	0.8129 (2)	0.8414 (2)	0.0339 (6)	
H2N	−0.0496	0.7755	0.8279	0.041*	
C1	0.2413 (6)	0.8436 (4)	0.5749 (3)	0.0640 (13)	
H1	0.2270	0.9068	0.5402	0.077*	
C2	0.3170 (7)	0.7649 (4)	0.5300 (4)	0.0749 (15)	
H2	0.3525	0.7736	0.4656	0.090*	
C3	0.3405 (6)	0.6719 (4)	0.5811 (4)	0.0653 (13)	
H3	0.3912	0.6162	0.5517	0.078*	
C4	0.2874 (5)	0.6627 (3)	0.6769 (3)	0.0465 (9)	
H4	0.3038	0.6013	0.7137	0.056*	
C5	0.2098 (4)	0.7455 (3)	0.7174 (3)	0.0359 (8)	
C6	0.1564 (4)	0.7408 (3)	0.8239 (3)	0.0378 (8)	
H6A	0.1276	0.6685	0.8394	0.045*	
H6B	0.2349	0.7601	0.8685	0.045*	
C7	0.0213 (4)	0.8512 (3)	0.9465 (3)	0.0371 (8)	
H7	0.1060	0.8960	0.9594	0.045*	
C8	−0.1117 (5)	0.9209 (4)	0.9566 (3)	0.0579 (11)	
H8A	−0.1047	0.9795	0.9110	0.087*	
H8B	−0.1180	0.9474	1.0236	0.087*	
H8C	−0.1967	0.8799	0.9415	0.087*	
C9	0.0207 (4)	0.7636 (3)	1.0248 (3)	0.0367 (8)	
C10	−0.0433 (4)	0.6656 (3)	1.0102 (3)	0.0439 (8)	
H10	−0.0836	0.6494	0.9485	0.053*	
C11	−0.0482 (5)	0.5911 (4)	1.0863 (3)	0.0546 (10)	
H11	−0.0918	0.5255	1.0753	0.065*	
C12	0.0112 (5)	0.6139 (4)	1.1780 (3)	0.0608 (13)	
H12	0.0074	0.5641	1.2293	0.073*	
C13	0.0767 (5)	0.7114 (4)	1.1932 (3)	0.0581 (12)	
H13	0.1183	0.7270	1.2547	0.070*	
C14	0.0804 (5)	0.7855 (4)	1.1175 (3)	0.0492 (10)	
H14	0.1237	0.8511	1.1287	0.059*	

### Atomic displacement parameters (Å^2^)

**Table:**

	*U*^11^	*U*^22^	*U*^33^	*U*^12^	*U*^13^	*U*^23^
Zn	0.0526 (3)	0.0350 (2)	0.0345 (2)	0.00634 (18)	0.00028 (19)	0.00188 (16)
Cl1	0.0638 (6)	0.0493 (5)	0.0448 (5)	0.0054 (5)	−0.0134 (5)	−0.0018 (4)
Cl2	0.0673 (6)	0.0459 (5)	0.0621 (6)	−0.0077 (5)	−0.0033 (5)	−0.0040 (5)
N1	0.056 (2)	0.0442 (17)	0.0388 (16)	0.0054 (16)	0.0066 (15)	0.0006 (14)
N2	0.0335 (14)	0.0380 (16)	0.0300 (14)	0.0003 (12)	−0.0007 (12)	−0.0015 (11)
C1	0.090 (3)	0.062 (3)	0.040 (2)	0.018 (3)	0.018 (2)	0.015 (2)
C2	0.107 (4)	0.076 (3)	0.042 (2)	0.022 (3)	0.023 (3)	0.008 (2)
C3	0.083 (3)	0.061 (3)	0.051 (3)	0.018 (3)	0.016 (3)	−0.009 (2)
C4	0.062 (2)	0.042 (2)	0.0356 (19)	0.0057 (19)	0.0047 (18)	−0.0014 (16)
C5	0.0412 (18)	0.0338 (16)	0.0327 (17)	−0.0011 (14)	0.0002 (15)	−0.0014 (14)
C6	0.0420 (19)	0.0367 (18)	0.0347 (18)	0.0029 (16)	0.0051 (16)	−0.0003 (15)
C7	0.0389 (18)	0.0400 (18)	0.0324 (17)	0.0009 (15)	0.0027 (15)	−0.0043 (14)
C8	0.068 (3)	0.058 (3)	0.048 (2)	0.025 (2)	0.011 (2)	0.006 (2)
C9	0.0335 (17)	0.046 (2)	0.0304 (17)	0.0053 (15)	0.0034 (15)	0.0004 (15)
C10	0.0431 (19)	0.047 (2)	0.0415 (19)	0.0011 (18)	0.0057 (18)	−0.0013 (16)
C11	0.060 (2)	0.048 (2)	0.056 (2)	0.006 (2)	0.011 (2)	0.0083 (19)
C12	0.065 (3)	0.071 (3)	0.047 (2)	0.027 (2)	0.012 (2)	0.017 (2)
C13	0.063 (3)	0.079 (3)	0.0326 (19)	0.015 (3)	−0.0036 (19)	0.000 (2)
C14	0.046 (2)	0.061 (2)	0.040 (2)	−0.0008 (19)	−0.0015 (18)	−0.0048 (19)

### Geometric parameters (Å, °)

**Table:**

Zn—N1	2.037 (3)	C6—H6B	0.970
Zn—N2	2.089 (3)	C7—C8	1.516 (5)
Zn—Cl1	2.2025 (12)	C7—C9	1.522 (5)
Zn—Cl2	2.2134 (11)	C7—H7	0.980
N1—C5	1.332 (5)	C8—H8A	0.960
N1—C1	1.338 (5)	C8—H8B	0.960
N2—C6	1.479 (4)	C8—H8C	0.960
N2—C7	1.492 (4)	C9—C10	1.380 (5)
N2—H2N	0.910	C9—C14	1.387 (5)
C1—C2	1.354 (7)	C10—C11	1.386 (6)
C1—H1	0.930	C10—H10	0.930
C2—C3	1.373 (7)	C11—C12	1.377 (7)
C2—H2	0.930	C11—H11	0.930
C3—C4	1.379 (6)	C12—C13	1.382 (8)
C3—H3	0.930	C12—H12	0.930
C4—C5	1.375 (5)	C13—C14	1.378 (6)
C4—H4	0.930	C13—H13	0.930
C5—C6	1.511 (5)	C14—H14	0.930
C6—H6A	0.970		
			
N1—Zn—N2	83.58 (12)	N2—C6—H6B	109.2
N1—Zn—Cl1	110.92 (11)	C5—C6—H6B	109.2
N2—Zn—Cl1	109.70 (9)	H6A—C6—H6B	107.9
N1—Zn—Cl2	113.45 (11)	N2—C7—C8	109.1 (3)
N2—Zn—Cl2	117.35 (9)	N2—C7—C9	114.7 (3)
Cl1—Zn—Cl2	117.13 (4)	C8—C7—C9	110.8 (3)
C5—N1—C1	118.4 (4)	N2—C7—H7	107.3
C5—N1—Zn	113.3 (2)	C8—C7—H7	107.3
C1—N1—Zn	127.9 (3)	C9—C7—H7	107.3
C6—N2—C7	113.6 (3)	C7—C8—H8A	109.5
C6—N2—Zn	107.5 (2)	C7—C8—H8B	109.5
C7—N2—Zn	113.7 (2)	H8A—C8—H8B	109.5
C6—N2—H2N	107.3	C7—C8—H8C	109.5
C7—N2—H2N	107.3	H8A—C8—H8C	109.5
Zn—N2—H2N	107.3	H8B—C8—H8C	109.5
N1—C1—C2	123.1 (4)	C10—C9—C14	118.3 (4)
N1—C1—H1	118.4	C10—C9—C7	123.4 (3)
C2—C1—H1	118.4	C14—C9—C7	118.2 (4)
C1—C2—C3	118.9 (4)	C9—C10—C11	120.9 (4)
C1—C2—H2	120.5	C9—C10—H10	119.5
C3—C2—H2	120.5	C11—C10—H10	119.5
C2—C3—C4	118.6 (4)	C12—C11—C10	120.2 (4)
C2—C3—H3	120.7	C12—C11—H11	119.9
C4—C3—H3	120.7	C10—C11—H11	119.9
C3—C4—C5	119.4 (4)	C11—C12—C13	119.4 (4)
C3—C4—H4	120.3	C11—C12—H12	120.3
C5—C4—H4	120.3	C13—C12—H12	120.3
N1—C5—C4	121.6 (3)	C14—C13—C12	120.2 (4)
N1—C5—C6	117.4 (3)	C14—C13—H13	119.9
C4—C5—C6	120.9 (3)	C12—C13—H13	119.9
N2—C6—C5	112.2 (3)	C13—C14—C9	121.0 (4)
N2—C6—H6A	109.2	C13—C14—H14	119.5
C5—C6—H6A	109.2	C9—C14—H14	119.5
			
N2—Zn—N1—C5	−2.7 (3)	C3—C4—C5—C6	−176.8 (4)
Cl1—Zn—N1—C5	−111.4 (3)	C7—N2—C6—C5	−152.2 (3)
Cl2—Zn—N1—C5	114.3 (3)	Zn—N2—C6—C5	−25.6 (3)
N2—Zn—N1—C1	169.4 (5)	N1—C5—C6—N2	26.0 (4)
Cl1—Zn—N1—C1	60.7 (5)	C4—C5—C6—N2	−157.8 (3)
Cl2—Zn—N1—C1	−73.5 (5)	C6—N2—C7—C8	−178.0 (3)
N1—Zn—N2—C6	15.9 (2)	Zn—N2—C7—C8	58.7 (4)
Cl1—Zn—N2—C6	125.8 (2)	C6—N2—C7—C9	−53.2 (4)
Cl2—Zn—N2—C6	−97.2 (2)	Zn—N2—C7—C9	−176.4 (2)
N1—Zn—N2—C7	142.5 (3)	N2—C7—C9—C10	−34.9 (5)
Cl1—Zn—N2—C7	−107.6 (2)	C8—C7—C9—C10	89.0 (4)
Cl2—Zn—N2—C7	29.3 (3)	N2—C7—C9—C14	148.8 (4)
C5—N1—C1—C2	1.7 (8)	C8—C7—C9—C14	−87.2 (4)
Zn—N1—C1—C2	−170.0 (4)	C14—C9—C10—C11	0.2 (6)
N1—C1—C2—C3	−0.9 (10)	C7—C9—C10—C11	−176.1 (4)
C1—C2—C3—C4	−0.8 (9)	C9—C10—C11—C12	−0.1 (6)
C2—C3—C4—C5	1.6 (8)	C10—C11—C12—C13	−0.5 (7)
C1—N1—C5—C4	−0.8 (6)	C11—C12—C13—C14	0.9 (7)
Zn—N1—C5—C4	172.1 (3)	C12—C13—C14—C9	−0.8 (7)
C1—N1—C5—C6	175.4 (4)	C10—C9—C14—C13	0.2 (6)
Zn—N1—C5—C6	−11.7 (4)	C7—C9—C14—C13	176.7 (4)
C3—C4—C5—N1	−0.8 (6)		
